# Effect of Sewage Sludge Biochar (SSB) on the Root Bacterial Community of Strawberry (*Fragaria × ananassa*): A 16S rRNA Gene Sequencing Approach

**DOI:** 10.3390/microorganisms14020319

**Published:** 2026-01-29

**Authors:** Erasmus Kabu Aduteye, Caleb Nindo, Ravendra P. Chauhan, Naveen Kumar Dixit

**Affiliations:** 1 Department of Agriculture, Food, and Resource Sciences, University of Maryland Eastern Shore, Princess Anne, MD 21853, USA; aekabu@umes.edu (E.K.A.); cinindo@umes.edu (C.N.); 2 Department of Recombinant Vaccines, Intercollegiate Faculty of Biotechnology, University of Gdansk, Abrahama 58, 80307 Gdansk, Poland; ravendra.chauhan@ug.edu.pl

**Keywords:** biochar, strawberry, 16S rRNA, soil health, alpha diversity, beta diversity, bacterial community, Cyanobacteriota

## Abstract

Strawberry (*Fragaria × ananassa*) is one of the most consumed berries worldwide. Despite improvements in management practices and breeding, maintaining soil health and minimizing environmental impact remain a challenge for agricultural systems. Biochar has been proposed as an effective strategy to mitigate climate change, enhance soil health, and promote plant growth. This study investigated the effects of sewage sludge biochar (SSB; 0% control, 5%, and 10% *w*/*w*) on the root-associated bacterial community of strawberry plants grown in pots under greenhouse conditions. Results obtained using 16S rRNA gene sequencing revealed a stable core bacterial community comprising 1207 amplicon sequence variants (ASVs), representing 13.2% of all detected ASVs and shared across all treatments. In contrast, biochar-amended soils harbored distinct sets of unique ASVs, with 1795 ASVs (19.7%) in the 5% SSB treatment and 2097 ASVs (23.0%) in the 10% SSB treatment, indicating treatment-specific community differentiation. Phylum-level analysis showed that Cyanobacteriota and Proteobacteria dominated the root-associated bacterial communities across all treatments, with no significant differences between biochar-amended soils and control groups. Alpha diversity did not differ significantly among treatments (*p* > 0.05), but beta diversity indicated subtle shifts in bacterial community composition under SSB amendment. SSB application increased community homogeneity, while overall bacterial diversity remained unchanged, indicating bacterial community restructuring rather than functional enhancement.

## 1. Introduction

Strawberry (*Fragaria × ananassa*) is a popular fruit crop, having gained significant interest in the past several decades, thus increasing demand for specialty crops [1]. Depending on the cropping system used, strawberry production can be either annual or perennial, and many improvements in management practices, breeding, and new systems have helped to support the commercial strawberry industry [2]. Achieving optimal strawberry production while simultaneously preserving soil health and reducing environmental impact continues to be a significant challenge in strawberry production [3]. In sustainable agriculture, soil health is vital to global ecosystem stability. It encompasses the soil’s physical, chemical, and biological attributes that collectively determine its capacity to support plant growth and maintain environmental quality. Soil health is a fundamental determinant of agricultural productivity, as soil physical, chemical, and biological properties, particularly rhizosphere microbial communities, regulate nutrient availability, root development, stress tolerance, and disease resistance, thereby directly influencing crop yield and quality [4,5]. Beyond agriculture, healthy soils contribute to various ecosystem services, such as water purification, carbon sequestration, and biodiversity conservation [6]. Biochar production and application have been proposed as an effective strategy to mitigate climate change and improve soil health [7]. Biochar is a charcoal-like substance that is made by burning organic material from agricultural and forestry wastes in a controlled process called pyrolysis [8]. Sewage sludge biochar (SSB) has attracted the attention of many researchers over the past decade due to its effectiveness in enhancing soil quality in agricultural fields. Sewage sludge is a semi-solid material generated as a byproduct during municipal or industrial wastewater treatment [9]. Along with other nutrients, it is a rich source of fertilizers, particularly potassium and phosphorus. Incorporating SSB into agricultural fields can be beneficial for advancing sustainable agriculture practices and systems.

Research is increasing on the effect of biochar on soil health focusing on the root microbial community, which plays a crucial role in plant growth, stress tolerance, and disease resistance. Studies have focused on the inherent properties of biochar, such as its high carbon content, structural stability preventing decomposition, large surface area, and porosity, which contribute to its ability to mitigate greenhouse gas emissions [10]. Fewer studies have explored the impact of biochar on the genetic profile of soil microbes and the structure of soil microbial communities, which serve as indicators of microbial biodiversity [11]. Ngaba and others [12] reported that biochar amendments can significantly alter soil microbial community and biomass. Biochar properties, such as particle size, and carbon composition, can influence soil microbial community structure and activity, although responses vary widely across soils, crops, and experimental conditions [13,14].

One potential explanation for biochar’s effect on root-associated bacterial community is its ability to alter the microbial community of the rhizosphere, facilitating propagation of beneficial microorganisms that stimulate plant growth and induce plant resistance [15]. This corresponds to accumulating evidence underlining the importance of soil- and root-associated microbiota in plant health [16]. Biochar’s capacity to enhance soil structure, nutrient availability, and water retention, which encourages beneficial microorganisms and increases disease resistance, creates a favorable environment for plant health. This promotes nutrient absorption, disease resistance, and plant resilience, resulting in higher agricultural yields where biochar application under field and greenhouse conditions for a variety of crops, including legumes such as soybean, has increased plant height, dry weight, and nutrient uptake [17]. These positive effects highlight the potential of biochar as a promising strategy for sustainable agriculture.

Despite extensive research on biochar effects on soil properties and plant performance, critical knowledge gaps remain regarding how different biochar application rates influence the root-associated bacterial community of strawberry plants at high taxonomic resolution. Most previous studies have focused on bulk soil or rhizosphere communities, plant- or manure-derived biochar [18], and field-scale agronomic outcomes [19], with limited attention to root-specific microbial responses or rate-dependent effects of sewage sludge-derived biochar (SSB). Moreover, few studies have applied amplicon sequence variant (ASV)-based 16S rRNA sequencing to resolve subtle microbial community reorganization within strawberry roots. This study addresses these gaps by systematically evaluating rate-dependent effects of SSB (0%, 5%, and 10% *w*/*w*) on the root-associated bacterial community of strawberry plants under controlled greenhouse conditions, thereby providing new mechanistic insight into biochar–bacterial community interactions in a specialty crop system.

## 2. Results

**Figure 1:**
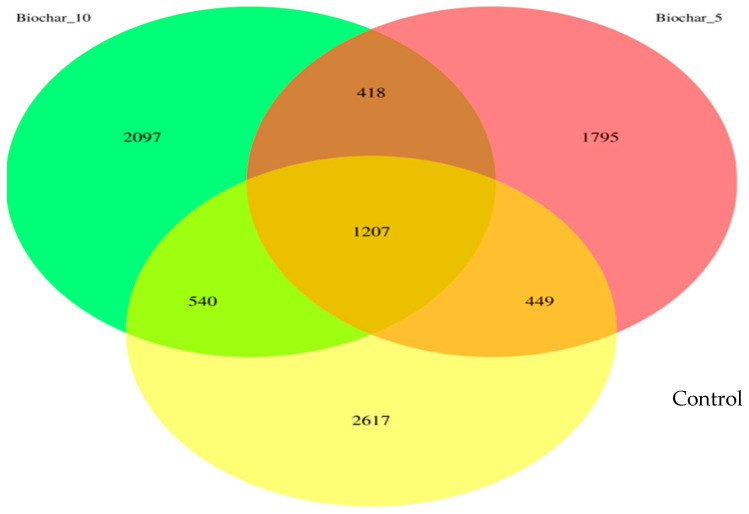
The Venn diagram illustrates the unique and shared amplicon sequence variants (ASVs). There are three biochar treatment groups shown in the Venn diagram: Control (no biochar), 5% SSB, and 10% SSB. The ASVs are unique and shared between these groups.

This study investigated the root-associated bacterial communities of “Seascape” a day-neutral strawberry grown in a greenhouse using SSB amendment. The results provide insights into how different SSB concentration amendments influence microbial community composition, with potential implications for plant–microbe interactions and soil functioning. A comprehensive view of the changes in microbial diversity and composition caused by the SSB amendments is presented in a Venn diagram (Figure 1). Biochar-amended soils exhibited distinct ASVs, with 1795 ASVs (19.7%) unique to the 5% SSB treatment and 2097 ASVs (23.0%) unique to the 10% SSB treatment, compared with 2617 ASVs (28.7%) unique to the control. These patterns indicate that SSB application modified niche availability and promoted treatment-specific bacterial assemblages without disrupting the shared core community.

The 10% SSB had a higher number of ASVs compared to the 5% treatment, suggesting a rate-dependent restructuring of microbial niches rather than a simple stimulation of microbial growth. A moderate SSB application (5%) may impose selective pressure that suppresses certain taxa; however, a higher amendment rate (10%) may create additional microsites and physicochemical heterogeneity that allow a broader range of microbial taxa to persist. The higher number of ASVs observed in the 10% SSB treatment does not indicate an overall increase in microbial diversity, as alpha diversity indices did not differ significantly among treatments. Rather, this pattern reflects changes in taxonomic composition and potential niche availability associated with biochar amendment. These compositional shifts occurred without evidence of enhanced richness or evenness, indicating microbial community restructuring rather than diversity enhancement [20].

The result highlighted the overlapping microbial populations among the treatments. Results revealed a stable core bacterial community comprising 1207 ASVs (13.2% of total detected ASVs) shared across all treatments. The fact that the core bacterial community stayed the same after the biochar was added suggests that some microbial species are very well adapted to the rhizosphere environment of the strawberries and are probably involved in important processes like plant growth, nutrient cycling, and the breakdown of organic matter. The 5% and 10% SSB treatments shared a total of 418 ASVs, indicating similar microbial responses to biochar application. There were fewer overlaps (449 ASVs) between the control and 5% SSB amendments and (540 ASVs) between the control group and 10% SSB amendment.

### 2.1. Stability of Microbial Community Structure at the Phylum Level

**Figure 2:**
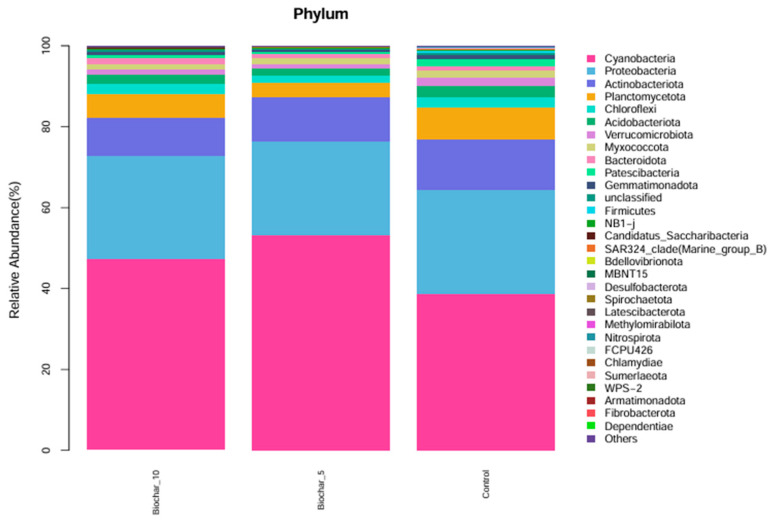
Relative abundance of microbial phyla under different SSB treatments. The stacked bar chart illustrates the relative abundance (%) of microbial phyla in the root-associated bacterial community of strawberry plants under different SSB treatments (10% SSB, 5% SSB, and control with no biochar). Each color represents a distinct microbial phylum, showing the proportion of each group within each treatment (*n* = 5 per treatment).

The relative abundance of microbial phyla across three treatments was studied (Figure 2). The bar plots represent the composition of microbial communities at the phylum level; different colors represent different phyla. Cyanobacteriota and Proteobacteria were the two most abundant phyla across all treatments, representing a major fraction of the microbial community within each group. Actinobacteriota, Planctomycetota, and Chloroflexota were also present, though their relative abundance was much lower than Cyanobacteriota and Proteobacteria. At the phylum level, the general composition of phyla appeared similar across the three treatments, indicating that, at this taxonomic level, the application of SSB at either 5% or 10% had minimal effect on microbial community structure. A variety of low-abundance phyla, such as Patescibacteriota, Gemmatimonadota, Firmicutes, and Spirochaetota, that contribute to diversity were also present, but in smaller proportions that did not alter the community structure in any treatment. The phylum composition of microbial community across treatments and the stability of major phyla relative abundance indicates a stable structure at the phylum level in response to 5% and 10% SSB application.

### 2.2. Impact of SSB on Microbial Community Composition at the Genus Level

**Figure 3:**
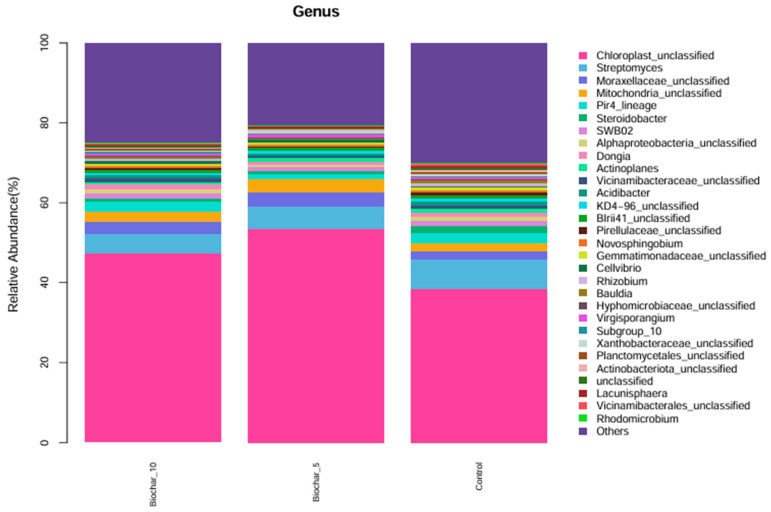
Relative abundance of microbial genera under different SSB treatments. The stacked bar plot illustrates the relative abundance (%) of microbial genera found in the root-associated bacterial community of strawberry plants under different SSB treatments: 10%, 5%, and control (no biochar). Each color represents a different microbial genus, showing their proportion within the total microbial community for each treatment (*n* = 5 per treatment).

The genus level taxonomic profile was dominated by sequences classified as Chloroplast_unclassified and Mitochondria_unclassified (Figure 3). These sequences originate from plant chloroplast and mitochondrial 16S rRNA genes and are a well-documented artifact of 16S rRNA amplicon sequencing in plant root tissues, where host organelle DNA is co-extracted and co-amplified with bacterial DNA. Although these reads do not represent true bacterial taxa, they were retained in taxonomic bar plots for transparency and to illustrate sequencing composition; however, they were excluded from downstream diversity, ordination, and statistical analyses to avoid bias in microbial community interpretation. Although not of bacterial origin, they were retained in taxonomic bar plots for transparency but excluded from downstream diversity and community composition analyses. Beyond chloroplast-derived reads, a considerable portion of the microbial community consisted of bacterial taxa that remained unclassified at the genus level. These included Moraxellaceae_unclassified, Vicinamibacteraceae_unclassified, Chitinophagaceae_unclassified, Oxalobacteraceae_unclassified, Xanthomonadaceae_unclassified, Sphingomonadaceae_unclassified, and Unclassified_Gammaproteobacteria. The consistent presence of these unclassified groups across all treatments suggests they are core members of the root-associated bacterial community, despite the limited resolution provided by current reference databases. Many of these families are known to harbor functionally important bacteria involved in nutrient cycling, organic matter degradation, and plant–microbe interactions. In contrast, classified genera such as *Rhizobium*, *Sphingomonas*, *Flavobacterium*, and *Variovorax* were observed at lower relative abundances. These genera are commonly associated with beneficial plant-associated traits, including nitrogen fixation, root colonization, and stress alleviation. Their presence, particularly under SSB treatments, may reflect shifts in microbial structure and function induced by organic amendments.

The community composition of 5% SSB and 10% SSB were similar (with Chloroplast_unclassified and *Streptomyces*). The control treatment also showed a similar profile, though some of the minor genera, such as *Dongia*, Pir4_lineage, and *Acidibacter*, were slightly different in relative abundance compared to SSB-treated samples. Overall, there was a similarity of dominant genera across all treatments, indicating that 5% and 10% application of SSB did not cause a significant change in microbial community structure at the genus level. The findings showed minor genus-level variations in relative abundance among treatments, most notably the genera found at lower levels in the control compared to SSB treatments. The high abundance of *Streptomyces* is remarkable, a genus that contributes to soil health through organic matter decomposition and potentially disease suppression. Its high abundance across treatments may indicate the presence of an efficient, stable functionality promoting a consistent benefit to the strawberry root-associated bacterial community.

### 2.3. Alpha Diversity of Root-Associated Bacterial Communities Under SSB Treatments

**Figure 4:**
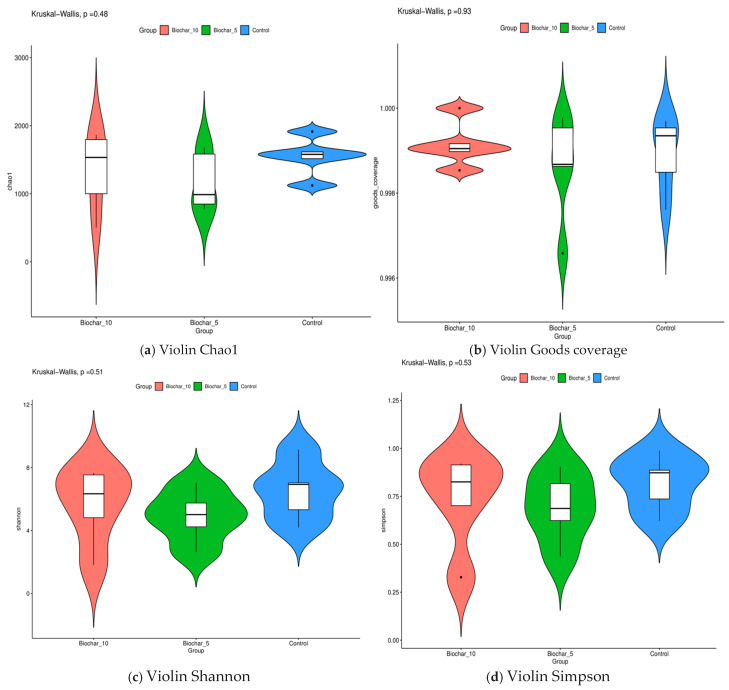
Alpha diversity metrics of root-associated bacterial communities under different sewage sludge biochar (SSB) treatments. (**a**) Chao1 richness index, reflecting estimated bacterial species richness. (**b**) Good’s coverage index indicates sequencing depth and sampling completeness. (**c**) The Shannon diversity index represents both species richness and evenness. (**d**) Simpson’s diversity index reflects dominance and community evenness. Violin plots show the distribution of each metric across treatments (10% SSB, 5% SSB, and control), with embedded boxplots indicating median and interquartile range. Each point represents an individual biological replicate (*n* = 5 per treatment). Statistical significance was assessed using the Kruskal–Wallis test; *p*-values are shown above each panel.

Using a variety of alpha diversity indices, we studied the changes in microbial communities. These included Chao1, Good’s coverage, Shannon, and Simpson indices that provide insights into richness, sampling completeness, and community structure across treatments. Alpha diversity metrics did not differ significantly among SSB treatments, indicating that biochar application altered community composition without substantially affecting overall bacterial richness or evenness (Figure 4). Using Kruskal–Wallis’s test, the result confirmed that there were no statistically significant differences between the treatments. The *p*-values for the Chao1, Good’s coverage, Shannon, and Simpson indices were 0.48, 0.93, 0.51, and 0.53 for each one. This finding implies that the application of SSB did not substantially impact the root-associated microbial diversity in strawberry plants. This wide coverage across treatments shows that the data we collected is accurate. Rarefaction curves indicated adequate sequencing depth across all samples, with curves approaching saturation, suggesting that the majority of bacterial diversity was captured (Supplementary Figures S2–S7). LEfSe (Linear Discriminant Analysis Effect Size) analysis further identified specific bacterial taxa significantly associated with biochar-amended treatments, indicating treatment-linked enrichment patterns (Supplementary Figure S1).

### 2.4. Beta Diversity

**Figure 5:**
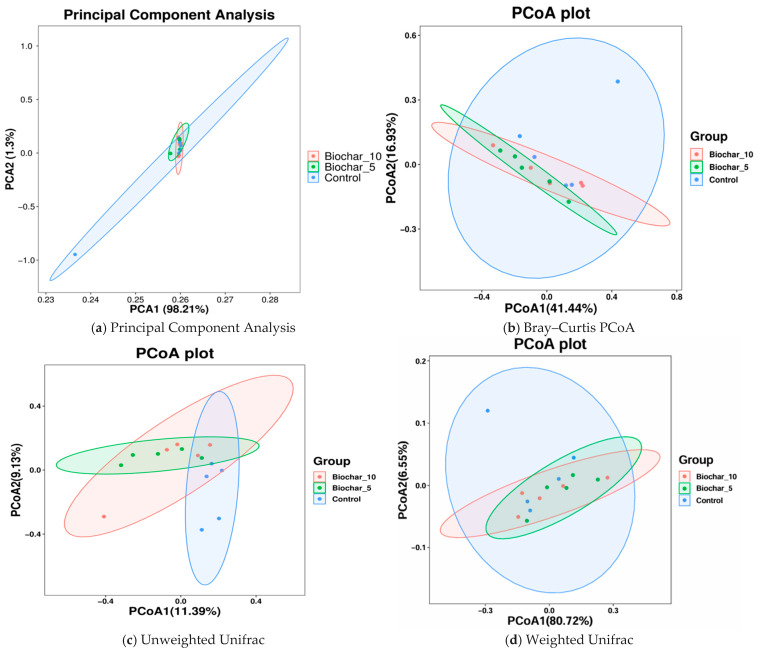
Ordination analysis of microbial community structure across SSB treatments. (**a**) Principal component analysis (PCA) showing variance in bacterial community composition among treatments based on abundance data. (**b**) Principal coordinates analysis (PCoA) based on Bray–Curtis dissimilarity, reflecting differences driven by relative abundances of taxa. (**c**) PCoA based on unweighted UniFrac distances, highlighting phylogenetic differences based on the presence or absence of taxa. (**d**) PCoA based on weighted UniFrac distances, incorporating both phylogenetic relationships and relative abundances. Each point represents an individual biological replicate (*n* = 5 per treatment), and ellipses indicate 95% confidence intervals. The percentage of variance explained by each axis is shown in parentheses.

In this study, both alpha diversity (within-sample diversity) and beta diversity (between-sample diversity) were evaluated to assess how SSB amendments influence microbial community structure. Phylum-level composition was broadly conserved across treatments, with dominant taxa remaining stable despite SSB application, suggesting compositional resilience at higher taxonomic levels (Figure 5). PCA1 and PCA2 represented 98.21% and 1.3% of the total variance, respectively (Figure 5a). PCA1 accounts for the most variation in the data and describes the highest-level differences among samples. Each color represents a single treatment sample, and ellipses represent 95% confidence intervals for each treatment group. Each of the three groups was densely clustered, with considerable overlap. This arrangement means that the microbial community composition of the treatments with SSB did not cause any substantial separation from the control. PCA2 shows that SSB has little effect on the main factors that cause differences in the composition of microbes, as shown by the high overlap and low variance. Microbial communities are likely similar across treatments, with no strong separation between SSB-treated and untreated (control) samples.

To test which factors in our experimental design contribute to differences in the beta diversity of microbial communities associated with the rhizosphere, PERMANOVA analyses were performed independently on each sample type using Bray–Curtis distances. To further visualize whether biochar amendment influenced microbial community composition, we conducted principal coordinate analysis (PCoA) using both Bray–Curtis and UniFrac distances. The PCoA plot was based on distance matrices, with PCoA1 and PCoA2 explaining 41.44% and 16.93% of the variance, respectively. Each treatment group has its confidence ellipse. The findings showed that the control group is more dispersed, suggesting a higher variability within as compared to the biochar-treated groups (Figure 5b). The ellipses of the biochar treatments (5% and 10% biochar) overlap with the control but are slightly closer to each other, indicating that biochar treatments could have a mild clustering effect. However, the overlap between all groups indicates no significant differences in microbial composition. There are signs of clustering in the biochar-treated samples, but the fact that the biochar treatments and the control samples are similar shows that biochar did not change the composition of the microbial communities. The moderate separation along PCoA1 (41.44% of variance) could be due to biochar’s small effects on community structure. Although the ordination plots may show only moderate separation, these subtle shifts can be ecologically and agriculturally meaningful.

Principal Coordinate Analyses of the weighted and unweighted UniFrac distance matrices (Figure 5c,d) were conducted to find the differences in the microbial community structure among the treatments. Unweighted UniFrac considers only the presence or absence of taxa and accounts for phylogenetic relationships. This plot indicates differences in community composition solely based on the phylogenetic diversity of taxa found per sample, irrespective of their relative abundances. Despite significant overlaps in the group distribution, PCoA1 shows some separation between the control and SSB groups. This separation is not particularly strong, indicating that while biochar treatment may change community composition compared to control, its overall effect on composition remains low. As shown in Figure 5c, PCoA1 and PCoA2 explained 11.39 and 9.33 percent of the variance, respectively. This finding suggests that simple presence-absence data may not fully explain significant differences in the makeup of communities. The Weighted UniFrac scores combine information about the phylogenetic history of organisms with information about how common they are. There is some overlap between the groups, but the control group is farther away from the SSB-treated groups along PCoA1 than PCoA2 (Figure 5d). The SSB-treated groups were more closely grouped, suggesting that these treatments have a similar impact on the structure of the microbial community, with the control group showing more variation. PCoA1 (80.72%) and PCoA2 (6.55%) plots show a larger portion of the variation. The result suggests that weighted Unifrac captured a lot of variation in community composition. While a statistical test such as PERMANOVA is important to confirm the significance of these differences, the visual evidence suggests that biochar influences the microbial community at varying concentrations. The absence of statistically significant differences in alpha diversity indices and PERMANOVA results indicates that SSB application did not strongly alter overall microbial diversity or community structure under the conditions tested.

**Figure 6:**
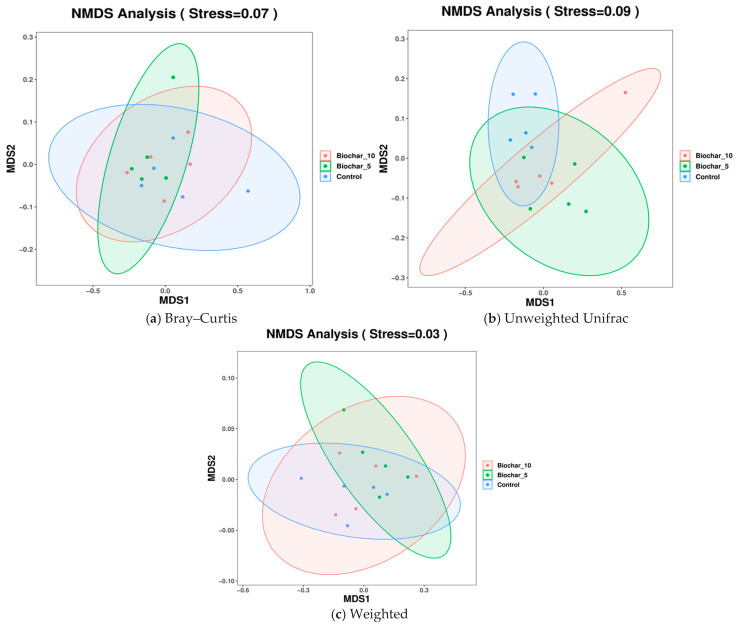
NMDS analysis of root-associated bacterial community composition under different sewage sludge biochar (SSB) treatments. (**a**) NMDS ordination based on Bray–Curtis dissimilarity, reflecting differences in bacterial community composition driven by relative abundance. (**b**) NMDS ordination based on unweighted UniFrac distances, highlighting phylogenetic differences based on the presence or absence of taxa. (**c**) NMDS ordination based on weighted UniFrac distances, incorporating both phylogenetic relationships and relative abundances of taxa. Each point represents an individual biological replicate (*n* = 5 per treatment), and ellipses indicate 95% confidence intervals. Stress values indicate the goodness of fit of the ordination, with lower values representing a more accurate two-dimensional representation.

Non-metric multidimensional scaling (NMDS) analyses revealed partial overlap among treatments across Bray–Curtis, unweighted UniFrac, and weighted UniFrac metrics, indicating subtle but treatment-associated shifts in microbial community composition (Figure 6). Each plot displays the microbial community composition across the three treatments. The stress values for all plots represent the quality of the NMDS analysis reflecting data into two-dimensional space, with the lower stresses indicating a more accurate representation. The NMDS based on Bray–Curtis (Stress = 0.07) considered the community composition differences based on taxa abundance, excluding the phylogenetic relationships (Figure 6a). The results were plotted with an overlap between the treatment groups and minor clustering of SSB treatments, and some separation of the control group from the other treatment groups towards the MDS1 axis. The composition of the community based on abundance was more similar in samples (roots) treated with SSB than in control samples. The clustering of biochar treatments indicates that the presence of biochar had some effect on the microbial abundance patterns to produce more homogeneous communities as compared to the control.

Unweighted UniFrac (Stress = 0.09) NMDS metric accounts for the presence or absence of taxa with respect to phylogenetic relationships (Figure 6b). The unweighted UniFrac plot had an overlap among the groups, but a slight separation was evident between the SSB-treated and control groups. A stress value of 0.09 indicates a reasonably good fit, not as strong as the Bray–Curtis plot. The overlap indicates that the application of SSB had minimal effect on the community composition (the absence or presence of taxa) between the treatments. This could indicate that biochar affected the abundance of selected taxa, such as Bray–Curtis, but not necessarily which taxa are found in each treatment group. The NMDS using Weighted UniFrac (Stress = 0.03) metric accounted for taxa abundance and phylogenetic relationships to reflect community composition, considering presence, abundance, and evolutionary associations among lineages (Figure 6c). The plot showed a more distinct clustering with separation between the control and SSB groups. The clustering of the SSB treatments (5% and 10%) is more evident, while the control samples are more apart. The clearer separation of the control from both SSB-treated samples indicates the influence on both microbial competition and phylogenetic structure or organic classification. The low stress value (0.03) indicates an ideal fit, and Weighted UniFrac shows an accurate representation of data.

### 2.5. Correlation Analysis

**Figure 7:**
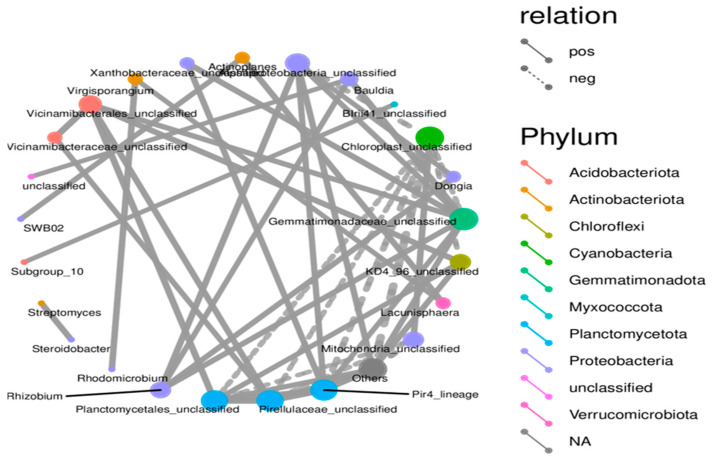
Microbial co-occurrence network depicting taxonomic interactions in SSB-amended strawberry root-associated bacterial community. The network graph represents the co-occurrence relationships among microbial taxa detected in the strawberry root-associated bacterial community across different SSB treatments, providing insights into microbial interactions under SSB amendment conditions.

There were links between microbes in the form of co-occurrence groups that have either a positive or negative relationship with genus or unclassified groups in different phyla (Figure 7). A node represents a specific microbial taxon (genus or unclassified group) and is annotated with its name. Node colors correspond to different phyla, shown in the color-coded legend on the right. Red nodes indicate taxa from Acidobacteriota, orange nodes represent Actinobacteriota, and blue nodes represent Planctomycetota. The size of the node is proportional to the relative abundance of that taxon, with larger nodes representing abundant taxa. Edges show connections between taxa, solid lines representing positive (co-occurrence) and dashed negative correlations (mutual exclusion or antagonistic relationship). The thicker the lines in the network, the stronger the correlation. The many solid lines connecting the taxa indicate their positive correlation. The result implies that these taxa are often present together, which could mean they help each other or share an ecological niche in the microbial community. The dashed lines showing a negative correlation indicate that if one taxon is present, another cannot be found. This synergistic effect suggests levels of competition or inhibition modes among these taxa.

The network analysis findings revealed a structured community with different cooperative and competitive interactions between microbes. Certain taxa are central to the network and likely contribute to both the stability and functional diversity of the community. Taxa with high connectivity, notably those within Planctomycetota and Chloroflexota, are potentially critical for different ecosystem functions. Their positive correlation with multiple other taxa indicates that they play a supportive or stabilizing role for these taxa in the microbial community. The application of SSB fosters a more diverse and interconnected microbial community that enhances beneficial microbial interactions and contributes to soil health and ecosystem stability.

## 3. Discussion

This research presents a characterization of the root-associated bacterial community of “Seascape” strawberry plants grown under greenhouse conditions and subjected to varying sewage sludge biochar (SSB) amendment rates. Strawberry roots with various SSB amendments were subjected to an in-depth study targeting the V3 and V4 regions of the 16S rRNA gene. This study specifically characterizes the root-associated bacterial community of strawberry plants rather than the bulk soil or rhizosphere community. In plant–microbe systems, the bulk soil, rhizosphere, and root-associated bacterial communities represent distinct ecological compartments shaped by different selective pressures. Bulk soil communities are primarily influenced by edaphic factors, whereas the rhizosphere is strongly structured by root exudates and nutrient gradients. In contrast, root-associated bacterial communities are further shaped by host-mediated selection processes, including immune filtering and tissue-specific colonization, resulting in more specialized microbial assemblages [21]. These distinctions are critical for interpreting microbial responses to biochar amendments and for comparing results across studies. Lazcano and others [22] demonstrated that strawberry rhizosphere microbial composition is linked to pathogen resistance and nutrient uptake under field conditions. In contrast, the present study examines root-associated microbial responses to SSB biochar under controlled greenhouse conditions, highlighting compartment and context-dependent bacterial community responses.

The 16S rRNA analysis of strawberry roots revealed that bacterial communities can be altered by SSB application in soil. The Venn diagram (Figure 1) showed a stable core bacterial community of 1207 ASVs present in all treatments. This showed the presence of important microbial taxa that are important for plant growth and ecosystem stability. The shared core bacterial community is an indication that some microbial taxa remained resilient after the application of SSB. These microbes may play a key role in nutrient cycling and the breakdown of organic matter. The root-associated bacterial community is considered to hold vast potential to support food production and plant-based industries. The core bacterial community is important for understanding the stable, consistent components across complex microbial assemblages. The unique ASVs observed in SSB-treated soils suggest that biochar modified the microbial niches, likely through changes in soil properties such as pH and water retention. While biochar is widely reported to influence soil pH, water-holding capacity, cation exchange capacity, and nutrient availability, these physicochemical properties were not directly measured in the present study. The microbial shifts observed in this study likely reflect biochar–bacterial community interactions at the community level; therefore, confirmation of specific physicochemical drivers will require integrated soil property measurements [23].

The lower ASVs observed in the 5% SSB amendment may be attributed to the selective pressure of the biochar on the soil microbes, altering the availability of nutrients and microenvironmental conditions (Figure 1). Root-associated microbial biodiversity also plays an important role in maintaining soil health and providing multiple functions and services simultaneously. Diverse soil communities are more efficient at breaking down organic matter and releasing nutrients. The control treatment exhibited 2617 unique ASVs, indicating a more diverse bacterial community in untreated soils. Despite the control exhibiting a greater number of unique ASVs, the SSB-amended soils may foster a more selective and functionally advantageous microbial community. Biochar can function as both a physical and chemical soil filter by modifying microbial habitats through its porous structure and reactive surface chemistry. The highly porous matrix of biochar provides protected microsites that support microbial colonization, while surface functional groups adsorb nutrients, organic compounds, and potentially inhibitory substances [24]. These combined properties can selectively favor certain microbial taxa while constraining others, thereby reshaping microbial community composition without necessarily increasing overall diversity. Such filtering effects are consistent with the observed rate-dependent shifts in ASV distribution following SSB application.

Beyond these mechanistic insights, the practical feasibility of applying higher rates of sewage sludge biochar at the field scale must be considered. Applying SSB at 10% (*w*/*w*) may be feasible in controlled greenhouse or pot experiments but presents economic and logistical challenges under field conditions due to material requirements, transportation, and application costs. While SSB may be more cost-effective based on biochar’s ability to valorize waste streams and offsetting wastewater disposal burdens, such high application rates are unlikely to be routinely practical in commercial production systems. In the present study, the 10% SSB treatment therefore serves as a mechanistic benchmark to elucidate rate-dependent microbial responses rather than as a recommended agronomic practice. These findings highlight the need for future field-scale studies integrating economic analyses, long-term soil health assessments, and optimized application strategies to identify cost-effective SSB rates for sustainable specialty crop production. While studies have reported that biochar amendments can enhance soil microbial diversity, the impact of biochar on microbial communities is complex and can vary depending on factors such as biochar type, application rate, soil properties, and environmental conditions [25]. In some cases, biochar application has been observed to reduce microbial diversity. A study conducted by Yang and others [26] reported that biochar addition enhanced the contents of environmentally persistent free radicals and derived hydroxyl radicals in the soil, while it reduced bacterial alpha diversity by 5.06–35.44%. The result from our study showed that control exhibited 2617 unique amplicon sequence variants, indicating a more diverse bacterial community compared to SSB-amended soils, which may be attributed to the specific conditions of the study, such as the type and amount of biochar used, soil characteristics, and the existing microbial community structure. This shift emphasizes the importance of microbial functionality and ecosystem services over mere species count in evaluating soil health [27].

Alpha diversity metrics (Chao1, Shannon, Simpson, and Good’s coverage) revealed no significant differences (*p* > 0.05) among treatments, suggesting that SSB altered the microbial composition; however, it did not substantially increase or decrease the overall diversity (Figure 4). This is likely due to biochar’s ability to improve soil structure, nutrient retention, and moisture availability, providing favorable conditions for a broader range of microbial species, including rare taxa. Inoculation with certain soil amendments can instead increase or not impact soil and rhizosphere alpha diversity, including application of biochar, vermicompost, a heavy metal immobilizer amendment, and bacterial inoculations [28]. A study by Fan and others [29] reported that the application of biochar influenced alpha-diversity indices (Shannon and Simpson) significantly after three years of biochar amendment. Similar studies were conducted by Wang and others [30] who reported that biochar addition changed soil diazotrophic abundance and community composition by changing the soil chemical and biological properties 3–4 years after biochar application in the rice field. The stability of alpha diversity index variations in diazotrophic community composition increased a few years after biochar application. The stable richness and evenness across treatments imply that biochar influences microbial community composition rather than species diversity [31]. The SSB application did not result in statistically significant changes in alpha diversity; however, a modest compositional shift was observed at certain taxonomic levels, suggesting a potential microbial reorganization rather than functional enhancement. Functional attributes such as nutrient cycling capacity or stress resilience were not directly measured in this study. Interpretations regarding microbial function were considered speculative and limited to compositional inferences based on taxonomic associations. Consistent with the absence of significant differences in alpha diversity, LEfSe analysis (Figure S1) revealed that SSB application primarily influenced the relative abundance of specific bacterial taxa rather than overall community richness. This suggests that biochar exerts selective pressures favoring certain taxa, leading to compositional reorganization without broad diversity shifts.

The beta diversity analyses (Figure 5 and Figure 6) indicated that SSB application caused subtle shifts in community composition but did not result in significant separation between treatments. The overlap observed in the PCA and PCoA plots suggests that SSB-treated soils retained microbial communities like those in control soils. However, the clustering of 5% and 10% SSB treatments in Weighted UniFrac (Figure 5d) and NMDS analyses (Figure 6a–c) indicate that SSB may have homogenized microbial communities. Such clustering might reflect biochar’s role in modifying the abundance of specific taxa rather than altering overall community composition. The moderate separation along PCoA1 in Weighted UniFrac analyses (Figure 5c) suggests that SSB treatments influenced the phylogenetic structure of the microbiome, possibly through selective enrichment of taxa better adapted to biochar-amended environments. It has been reported that the structure of the root-associated bacterial community could be affected by biochar application [32]. After six years of biochar application, a clear and considerable difference in bacterial community structure was observed between biochar-added and non-biochar-added soils [29]. Biochar amendment not only changed the soil parameters but also induced bacterial community separation in the soil.

The relatively consistent abundance of Cyanobacteriota and Proteobacteria across all treatments indicates phylum-level compositional stability in the strawberry root-associated bacterial community. Given the broad functional and taxonomic diversity encompassed within Proteobacteria, this observation reflects overall structural consistency rather than uniform functional responses, which are more appropriately resolved at lower taxonomic levels (Figure 2). These phyla play a crucial role in nitrogen fixation, organic matter decomposition, and plant–microbe interaction. Proteobacteria encompass an enormous level of morphological, physiological, and metabolic diversity, and are of great importance to global carbon, nitrogen, and sulfur cycling [33]. Proteobacteria and Firmicutes were reported as good indicators for reflecting changes in the main microbial groups and an important resource for exploring halophilic enzymes and metabolic pathways for pollutant remediation in saline soil. The slight shifts in minor phyla such as Acidobacteriota, Verrucomicrobiota, and Bacteroidota suggest that SSB may have specific, localized effects on less abundant taxa. At the genus level, the consistent abundance of *Streptomyces* across all treatments highlights its potential role in maintaining soil health. The ability of *Streptomyces* to decompose organic matter and suppress pathogens may contribute to biochar’s beneficial effects on plants [34]. The *Streptomyces* genus, which is the most abundant and arguably the most important actinomycete, is a good source of bioactive compounds, antibiotics, and extracellular enzymes [35]. These genera have shown, over time, great potential in improving the future of agriculture. The slight variations in low-abundance genera like *Dongia* and Pir4_lineage indicate that SSB might selectively promote or inhibit specific taxa, warranting further investigation into their ecological roles. At the genus and species levels, several taxa showed variation across treatments; however, interpretation of these patterns is constrained by taxonomic resolution and sequencing artifacts commonly associated with plant root samples. Functional roles discussed in this study are inferred from taxonomic affiliations and the existing literature rather than directly measured functional activity. Interpretations related to ecological processes such as nutrient cycling or plant stress responses were considered putative and hypothesis-generating, pending validation through functional assays or omics-based approaches. The dominance of chloroplast- and mitochondria-derived sequences at lower taxonomic levels highlights a common limitation of 16S rRNA gene sequencing in plant root systems and reinforces the need to interpret genus-level profiles with caution, focusing instead on higher taxonomic resolution and community-level patterns.

The microbial co-occurrence network revealed complex interactions, with both cooperative and antagonistic relationships. The predominance of positive correlations indicates patterns of co-occurrence among taxa; however, such associations do not necessarily reflect direct ecological interactions or functional cooperation [36]. Negative correlations, on the other hand, likely represent competition for limited nutrients, antagonistic metabolite production, or niche differentiation, which help maintain balance by preventing dominance of specific groups. Taxa with high connectivity, particularly those from Planctomycetota and Chloroflexota, play an important role in the network, such as stabilizing and linking multiple groups. Their strong positive correlations suggest they facilitate nutrient cycling and support other beneficial microbes, thereby contributing to soil ecosystem stability. Studies have reported that biochar amendments can exert both positive and negative effects on soil- and root-associated microbial communities, depending on biochar type, application rate, and environmental context [37]. Although several studies have reported biochar-induced shifts in soil or rhizosphere bacterial communities [38], these systems differ fundamentally from root-associated microbial communities, which are subject to stronger host selection and compartmentalization [39]. Collectively, biochar application appears to restructure microbial co-occurrence patterns, indicating potential shifts in community organization, although functional consequences and direct interactions were not assessed in this study.

## 4. Materials and Methods

### 4.1. Study Design

A greenhouse pot experiment was conducted in a randomized complete block design (RCBD) with three SSB treatments—control (0% *w*/*w*), 5% (*w*/*w*), and 10% (*w*/*w*)—using the “Seascape” strawberry cultivar. The experiment included a total of 60 plants (3 treatments × 5 replications × 4 pots per replication; one plant per pot). To reduce sequencing cost, microbial community analysis was conducted on a subset of plants. Specifically, one plant was randomly selected from each replication within each treatment, resulting in 15 independently sampled plants (*n* = 5 per treatment). Each sampled plant, grown in an individual pot, was treated as an independent biological replicate for downstream bacterial community analysis. Pots were randomly assigned to treatments and periodically repositioned within the greenhouse to minimize spatial effects. All root samples were collected at the same plant developmental stage to reduce temporal variability. Bare-root strawberries were bought from Indiana Berry and Plant Co. (Plymouth, IN, USA) and transplanted into 6.4 L pots in the second week of May 2023. Pots were kept in a greenhouse in natural light conditions (700–750 µmol m^−2^ s^−1^ photosynthetically active radiation (PAR)). The PAR reached approximately 400 µmol m^−2^ s^−1^ (Spectrum Technologies, Inc., Aurora, IL, USA) on a cloudy day. The greenhouse was about 27 ± 2 °C, 75% relative humidity, and a 12 h photoperiod was used. Plants were fertilized using a complete water-soluble mineral fertilizer (Peters Professional, 20–20–20; ICL Specialty Fertilizers, Allentown, PA, USA), supplying nitrogen (N), phosphorus (P), and potassium (K). Nitrogen was provided as a mixture of urea nitrogen (10.4%), nitrate nitrogen (5.5%), and ammoniacal nitrogen (4.1%), while phosphorus and potassium were supplied as P_2_O_5_ (20%) and K_2_O (20%), respectively. The fertilizer also contained essential micronutrients (Fe, Mn, Zn, Cu, B, and Mo). Fertilization began two weeks after transplanting and was applied as a nutrient solution at a rate of 1.25 g L^−1^ every 14 days. The same fertilization regime was applied uniformly across all treatments throughout the experiment. Plants were monitored regularly, and runners were removed weekly.

#### Biochar Source and Characteristics

The SSB used in this study was a commercially produced, International Biochar Initiative (IBI) certified biochar obtained from Wakefield Biochar (Bioforcetech Corporation, South San Francisco, CA, USA). The biochar was derived from municipal sewage sludge and produced under controlled pyrolysis conditions. On a dry-weight basis, the biochar exhibited high alkalinity (pH 11.32), high ash content (60.4%), and moderate organic carbon content (32.5%), with a hydrogen-to-carbon (H:C) molar ratio of 0.57 and an oxygen-to-carbon (O:C) ratio of 0.04, indicating a highly carbonized and chemically stable material (Table 1). The biochar had a specific surface area of 158 m^2^ g^−1^, a bulk density of 956.3 kg m^3^, and an electrical conductivity of 0.73 dS m^−1^, whereas the total nitrogen content was 3.59% (dry mass).

Elemental analysis revealed substantial concentrations of phosphorus, about 119.554 g kg^−1^, and potassium 7.874 g kg^−1^, while trace elements, including As, Cd, Pb, Hg, and Ni were within ranges reported for sewage sludge-derived biochar used in soil applications. Particle size distribution indicated that most biochar particles were <2 mm in diameter. These SSB physicochemical properties indicate a high-ash, alkaline biochar with substantial surface area and nutrient content. These characteristics are known to influence soil physicochemical conditions and microbial microhabitats.

### 4.2. Sample Collection

Strawberry samples were collected randomly after 180 days of transplanting, and the roots were used for the analysis. About 500 mg of fresh strawberry roots were transferred to a 15 mL centrifuge tube and prepared for shipment. The tubes were sealed with parafilm, then placed in about 5.5 kg of dry ice and packaged in a thermally insulated shipping box to prevent microbial degradation. To prevent the tubes from being crushed by ice packs during the shipping process, the 15 mL tubes were placed into a 50 mL centrifuge tube (Fisher Scientific, Houston, TX, USA, Polypropylene). The sample was shipped by overnight carrier to LC Sciences LLC (Houston, TX, USA) for 16s rRNA analysis. The fresh strawberry roots were shipped with careful attention to proper handling and transportation within a cold chain.

### 4.3. Experimental Method

Bacterial community composition was analyzed using 16S rRNA gene amplicon sequencing [22]. Amplicon libraries targeting the V3–V4 regions of the bacterial 16S rRNA gene were prepared using primers 341F and 805R. Sequencing was performed by LC Sciences (Houston, TX, USA) using Illumina sequencing-by-synthesis technology on a NovaSeq platform (San Diego, CA, USA), generating paired-end reads (2 × 250 bp). Raw sequence data were provided in FASTQ format and subjected to quality filtering, read merging, and chimera removal before downstream bioinformatic analyses. High-quality clean reads were denoised using the DADA2 algorithm to generate amplicon sequence variants (ASVs) at single-nucleotide resolution. This study focused on bacterial communities because they are the most abundant and functionally diverse root-associated bacterial communities that play a central role in nutrient cycling and plant–microbe interactions.

### 4.4. Bioinformatics Analysis

**Figure 8:**
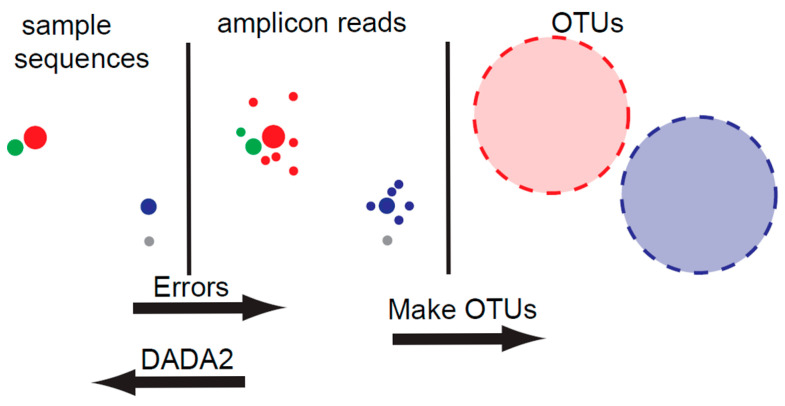
DADA2 Workflow for Error Correction and OTU Generation in Microbial Sequencing.

Sequence data processing and ASV inference were conducted by LC Sciences using the DADA2 algorithm within their standardized QIIME 2 bioinformatics pipeline. The exact QIIME 2 release and DADA2 plugin versions were not specified in the analytical report provided by the service provider (Figure 8). After quality filtering, read merging, and chimera removal, DADA2 was used to infer amplicon sequence variants (ASVs) at single-nucleotide resolution. This approach avoids arbitrary similarity thresholds associated with OTU clustering and provides higher taxonomic resolution and reproducibility. Quantitative Insights into Microbial Ecology 2 (QIIME 2) was used for analyzing and interpreting the data. The shared and unique ASVs were presented in a Venn diagram. Following taxonomic assignment, sequences classified as Chloroplast and Mitochondria were identified as host-derived organelle reads. These sequences were retained solely for initial taxonomic profiling and visualization to illustrate sequencing composition but were removed before all downstream analyses, including alpha diversity, beta diversity, ordination (PCoA and NMDS), and statistical testing. Accordingly, all diversity metrics and community structure analyses were performed exclusively on bacterial ASVs after exclusion of host-derived reads.

### 4.5. Bioinformatics Processing and Diversity Analysis

Raw paired-end FASTQ reads generated from NovaSeq sequencing were processed by LC Sciences (Houston, TX, USA). Reads were merged, quality filtered, and screened for chimeras to obtain high-quality sequences. Amplicon sequence variants (ASVs) were inferred using the DADA2 denoising algorithm, and an ASV feature table was generated for downstream analyses. Alpha diversity was calculated using observed features (ASV richness), Chao1, Shannon, Simpson, Good’s coverage, and Pielou’s evenness. Beta diversity was assessed using weighted and unweighted UniFrac, Bray–Curtis, and Jaccard distance matrices, with community differences visualized by PCA, PCoA, NMDS, and UPGMA clustering. Group-level differences in community composition were tested using ANOSIM and PERMANOVA (Adonis). PCA and NMDS analyses were conducted in R using the vegan package as part of the LC Sciences standardized bioinformatics workflow; however, the specific R and vegan package versions were not reported by the service provider.

### 4.6. Taxonomy Community

SILVA (Release 138, https://www.arb-silva.de/documentation/release-138/ accessed on 15 October 2025) and the NT-16S database were used for taxonomy, with the confidence level set at >0.7. Based on the ASV annotation, the abundance and differential results at different levels, including domain, phylum, class, order, family, genus, and species, were determined. Visual display of data at the phylum and genus levels was considered for interpretation. The top 30 taxa were selected for plotting with a stacked bar chart or heat map (clustered according to similarity) for each sample.

### 4.7. Statistical Analysis

Statistical analyses were performed by LC Sciences (Houston, TX, USA) using their standardized bioinformatics and statistical workflow. Differences in alpha diversity indices among treatments were evaluated using the Kruskal–Wallis test. Differences in community composition (beta diversity) were assessed using analysis of similarities (ANOSIM) and permutational multivariate analysis of variance (PERMANOVA; Adonis) based on distance matrices. Ordination analyses were used for visualization only. Statistical significance was determined at *p* < 0.05.

## 5. Conclusions

Sewage sludge biochar amendment has resulted in a slight change in the root-associated bacterial community of strawberry plants. A stable core bacterial community was maintained across all treatments. Nevertheless, a unique microbial taxon was observed in the SSB-amended soils. The alpha diversity measurements showed no significant differences, indicating that SSB application did not substantially alter overall bacterial richness or evenness. Beta diversity analyses revealed subtle, rate-dependent shifts in community composition, suggesting microbial reorganization rather than large-scale restructuring. Cyanobacteriota and Proteobacteria were the dominant microbial taxa across all treatments at the phylum level, with no significant differences between SSB-amended and control. The Chao1, Shannon, Simpson, and Good’s Coverage Alpha diversity indices showed no significant differences between treatments. The beta diversity showed a slight shift in microbial community composition in the SSB amendments. The 10% SSB treatment served as a mechanistic benchmark to explore rate-dependent microbial responses rather than as a recommended agronomic practice, given potential economic and logistical constraints at the field scale. These findings demonstrate that SSB application can subtly modulate the structure of the strawberry root-associated bacterial community under controlled conditions, while preserving community stability. Future research integrating soil physicochemical measurements, functional assays, and field-scale validation will be essential to determine the agronomic relevance and long-term implications of SSB amendments for sustainable strawberry production.

## Figures and Tables

**Table 1:** Physicochemical properties of the biochar used in the experiment.

Parameter	Value
pH	11.32
Organic carbon	32.5 (% dry mass)
Total nitrogen	3.59 (% dry mass)
Total ash	60.4 (% dry mass)
Hydrogen/Carbon (H:C)	0.57 (molar ratio)
Oxygen/Carbon (O:C)	0.04 (molar ratio)
Surface area	158 (m^2^ g^−1^)
Electrical conductivity	0.73 (dS m^−1^)
Total phosphorus	119.554 (g kg^−1^)
Total potassium	7.874 (g kg^−1^)

**Source:** Wakefield Biochar (Bioforcetech Corporation, South San Francisco, CA, USA).

## Data Availability

The original contributions presented in this study are included in the article/Supplementary Materials. Further inquiries can be directed to the corresponding author.

## Supplementary Materials

The following supporting information can be downloaded at https://www.mdpi.com/article/10.3390/microorganisms14020319/s1, Figure S1: LEfSe analysis identifying bacterial taxa differentially enriched between Control and 5% sewage sludge biochar (SSB) treatments in strawberry root-associated bacterial communities title; Figure S2: Good’s coverage curves of bacterial communities across biochar treatments and control as a function of sequencing depth; Figure S3: Sequencing depth versus observed ASV richness in biochar-treated and control bacterial communities; Figure S4: Sequencing depth versus Pielou’s evenness in biochar-treated and control bacterial communities.; Figure S5: Rarefaction curves of Shannon diversity index for bacterial communities under biochar treatments and control as a function of sequencing depth; Figure S6: Rarefaction curves of Simpson diversity index for bacterial communities under biochar treatments and control as a function of sequencing depth; Figure S7: Rarefaction curves of Chao1 richness estimator for bacterial communities under biochar treatments and control as a function of sequencing depth.
