# Supplementary material for: Effect of Sewage Sludge Biochar (SSB) on the Root Bacterial Community of Strawberry (Fragaria × ananassa): A 16S rRNA Gene Sequencing Approach
Source: Microorganisms. 2026 Jan 29;14(2):319. doi: 10.3390/microorganisms14020319 (PMC12943253; doi:10.3390/microorganisms14020319)
Supplement: Supplementary file 1 [file microorganisms-14-00319-s001.zip › microorganisms-4075187-supplementary.pdf]

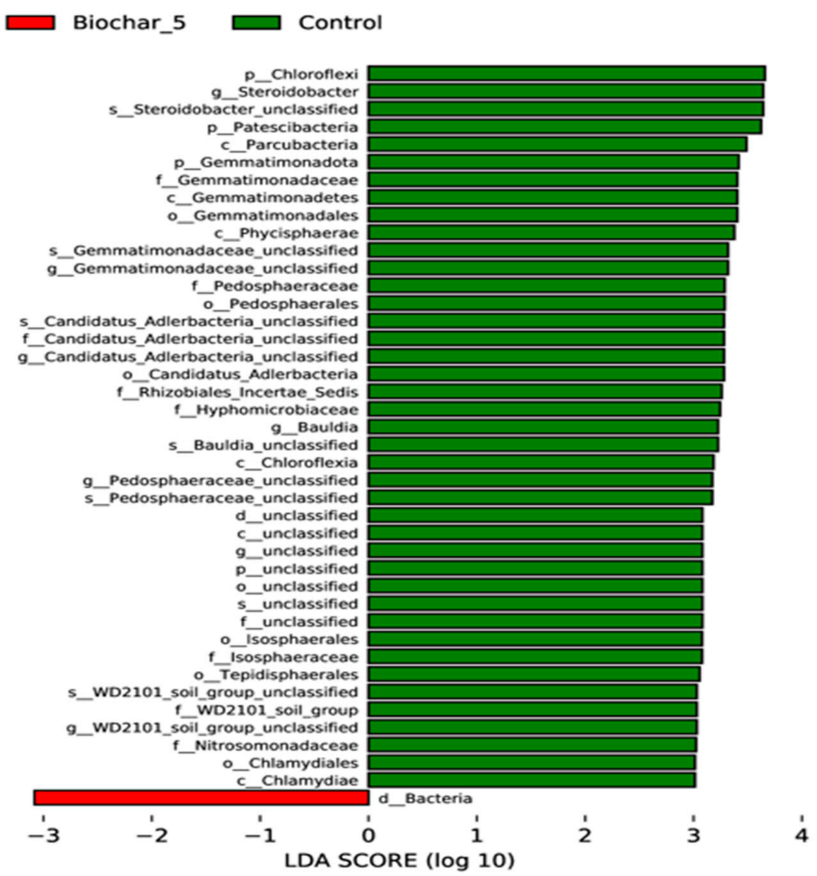

Figure S1. LEfSe analysis identifying bacterial taxa differentially enriched between Control and 5% sewage sludge biochar (SSB) treatments in strawberry root-associated bacterial communities.

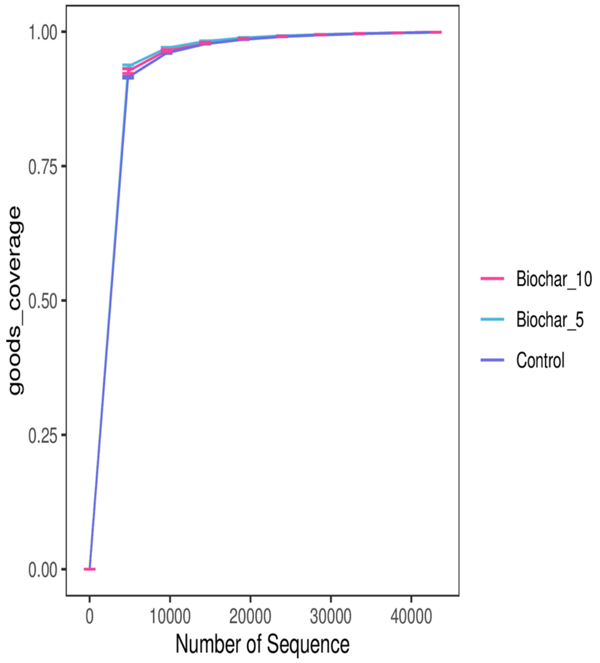

Figure S2. Good's coverage curves of bacterial communities across biochar treatments and control as a function of sequencing depth

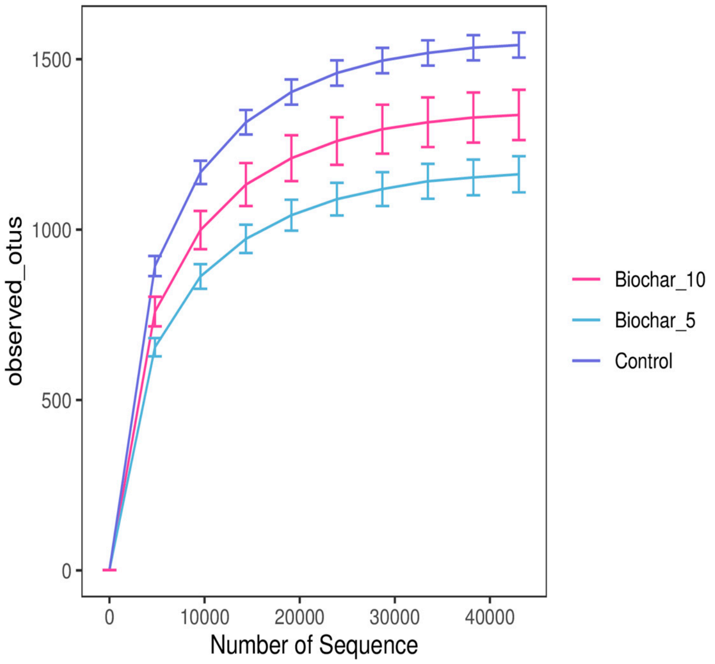

Figure S3. Sequencing depth versus observed ASV richness in biochar-treated and control bacterial communities

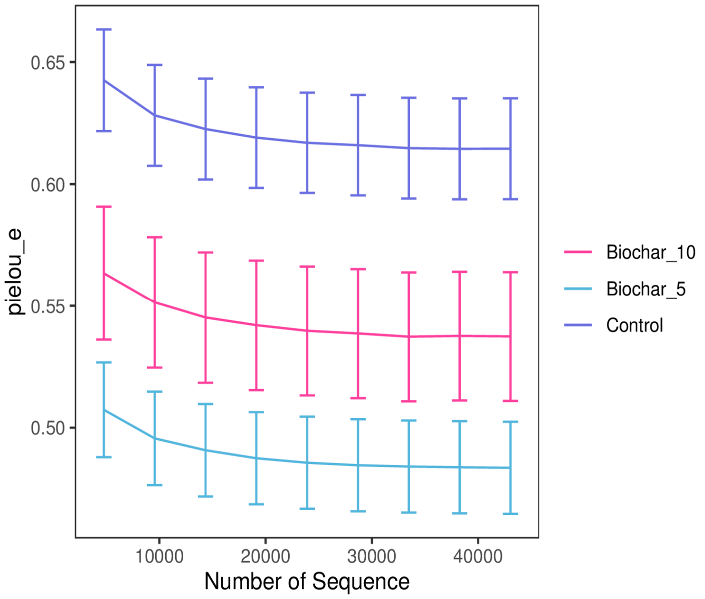

Figure S4. Sequencing depth versus Pielou's evenness in biochar-treated and control bacterial communities.

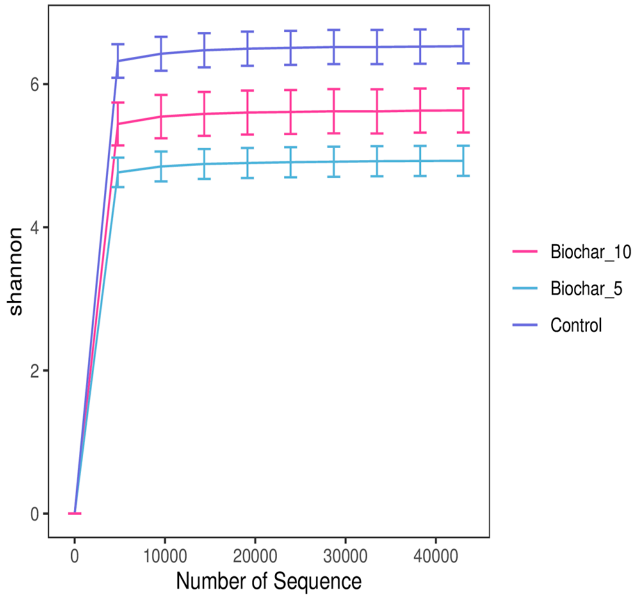

Figure S5. Rarefaction curves of Shannon diversity index for bacterial communities under biochar treatments and control as a function of sequencing depth

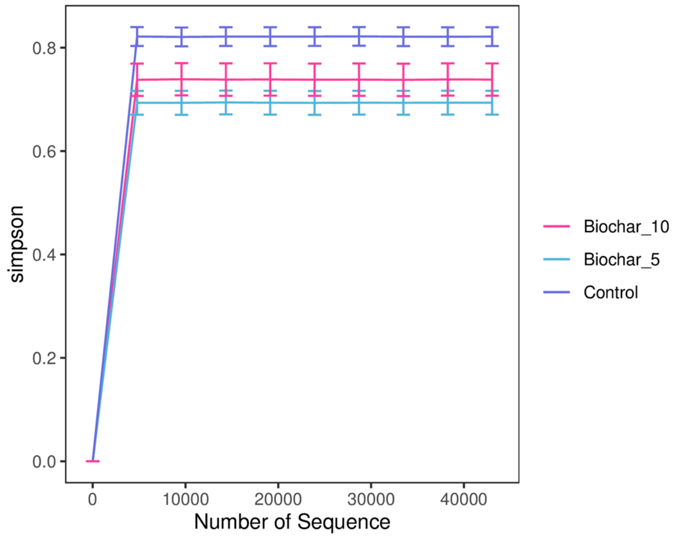

Figure 6. Rarefaction curves of Simpson diversity index for bacterial communities under biochar treatments and control as a function of sequencing depth.

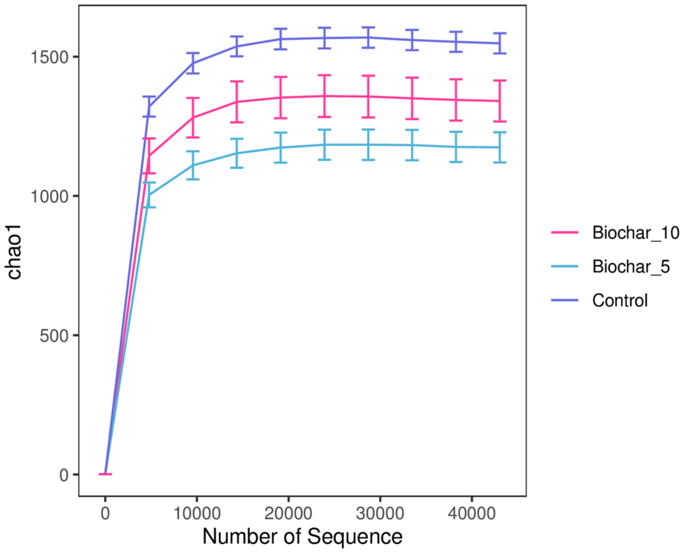

Figure S7. Rarefaction curves of Chao1 richness estimator for bacterial communities under biochar treatments and control as a function of sequencing depth.
